# Cancer Prevalence Across Vertebrates

**DOI:** 10.21203/rs.3.rs-3117313/v1

**Published:** 2023-07-06

**Authors:** Zachary T. Compton, Valerie Harris, Walker Mellon, Shawn Rupp, Diego Mallo, Stefania E. Kapsetaki, Mallory Wilmot, Ryan Kennington, Kathleen Noble, Cristina Baciu, Lucia Ramirez, Ashley Peraza, Brian Martins, Sushil Sudhakar, Selin Aksoy, Gabriella Furukawa, Orsolya Vincze, Mathieu Giraudeau, Elizabeth G. Duke, Simon Spiro, Edmund Flach, Hannah Davidson, Ashley Zehnder, Trevor A. Graham, Brigid Troan, Tara M. Harrison, Marc Tollis, Joshua D. Schiffman, Athena Aktipis, Lisa M. Abegglen, Carlo C. Maley, Amy M. Boddy

**Affiliations:** 1 Arizona Cancer Evolution Center, The Biodesign Institute, Arizona State University, Tempe, AZ; 2 School of Life Sciences, Arizona State University, Tempe, AZ; 3 Department of Pediatrics and Huntsman Cancer Institute, University of Utah, Salt Lake City, UT; 4 Department of Psychology, Arizona State University, Tempe, AZ; 5 Genomic Sciences Graduate Program, North Carolina State University, Raleigh, NC; 6 Institute of Aquatic Ecology, Centre for Ecological Research, 4026 Debrecen, Hungary; 7 Evolutionary Ecology Group, Hungarian Department of Biology and Ecology, Babeş-Bolyai University, 400006 Cluj-Napoca, Romania; 8 LIENSs, UMR 7266 CNRS-La Rochelle Université, La Rochelle, France,; 9 North Carolina State College of Veterinary Medicine, Raleigh, NC; 10 Exotic Species Cancer Research Alliance, North Carolina State College of Veterinary Medicine, Raleigh, NC; 11 Wildlife Health Services, Zoological Society of London, London, UK.; 12 Centre for Evolution and Cancer, Institute of Cancer Research, London, UK; 13 The North Carolina Zoo, Asheboro, NC; 14 School of Informatics, Computing and Cyber Systems, Northern Arizona University, Flagstaff, AZ; 15 Peel Therapeutics, Inc., Salt Lake City, UT; 16 Biodesign Center for Biocomputing, Security and Society, Arizona State University, Tempe, AZ; 17 University of California Santa Barbara, Santa Barbara, CA

## Abstract

Cancer is pervasive across multicellular species, but what explains differences in cancer prevalence across species? Using 16,049 necropsy records for 292 species spanning three clades (amphibians, sauropsids and mammals) we found that neoplasia and malignancy prevalence increases with adult weight (contrary to Peto’s Paradox) and somatic mutation rate, but decreases with gestation time. Evolution of cancer susceptibility appears to have undergone sudden shifts followed by stabilizing selection. Outliers for neoplasia prevalence include the common porpoise (<1.3%), the Rodrigues fruit bat (<1.6%) the black-footed penguin (<0.4%), ferrets (63%) and opossums (35%). Discovering why some species have particularly high or low levels of cancer may lead to a better understanding of cancer syndromes and novel strategies for the management and prevention of cancer.

## One Sentence Summary

Body weight, gestation time, and somatic mutation rates explain part of the variation in cancer prevalence across species.

## Introduction

Cancer is a ubiquitous problem for multicellular species ^1^ and a leading cause of death in humans ^2^. Every multicellular body is a cooperative cellular system, with cells suppressing replication^3^, dividing labor ^4^, sharing resources ^5^, regulating cell death ^6^ and taking care of the extracellular environment ^1^. However, cooperative systems are susceptible to cheaters, which emerge as cancers in multicellular organisms ^7^. Because cancer cells can outcompete normal cells with respect to replication, survival, resource use and other cellular behaviors, natural selection within the body can favor cancer cells via somatic evolution.

Cancer has been a strong selective pressure on multicellular organisms and mechanisms for cancer suppression likely co-evolved along with the evolution of multicellularity ^8,9^. Despite this persistent selective pressure of cancer, species vary in their investment in cancer defenses across the tree of life. Sir Richard Peto predicted in 1977 that the risk of cancer should scale with the number of cells in an organism and the length of its lifespan ^10^. This prediction is based on the fact that tumors evolve from single cells, partially due to the accumulation of somatic mutations over time ^10^. His observation that cancer risk does not appear to increase with increases in body mass and longevity across species ^10^, a phenomenon known as ‘Peto’s paradox’, launched the field of comparative oncology ^11^.

Early work in comparative oncology found that birds, and to a lesser extent reptiles, develop fewer neoplasms than mammals ^12–14^. While single case studies have been reported ^15^, it has been difficult to estimate true neoplasia prevalence in these taxa. In 2015, we published neoplasia prevalence estimates in 37 mammals and reported support for Peto’s Paradox, that is, bigger, longer-lived species do not get more cancer ^16^. Follow up studies have supported Peto’s Paradox and demonstrated the ubiquity of cancer across mammals ^17,18^. The extensive variation in cancer risk across vertebrates provides a unique opportunity to identify species with exceptional cancer resistance that can lead to new discoveries of cancer resistance mechanisms outside the traditional human and murine studies. Additionally, the discovery of cancer vulnerable species could lead to new insights into cancer syndromes as well as provide spontaneous ‘natural’ animal models of disease that can help us gain a better understanding of various types of cancer and their treatments. Here we present a large, curated database of tetrapod veterinary necropsy records, including 16,049 individual animals across 292 species of animals, encompassing reptiles, birds, amphibians, and mammals. Because necropsies typically are diagnosed with “neoplasia” which includes both benign and malignant tumors, we developed a terminology dictionary to distinguish benign from malignant neoplasms in the necropsy reports. We calculate and analyze both neoplasia prevalence as well as malignancy (cancer) prevalence. Only a subset of benign neoplasms evolve into cancers over a lifetime, so neoplasia prevalence is always greater than or equal to malignancy prevalence. We also tested for age bias in the animals that died with neoplasias or cancers.

## Results

### Variation in Cancer Risk Across Clades

**Fig. 1.**
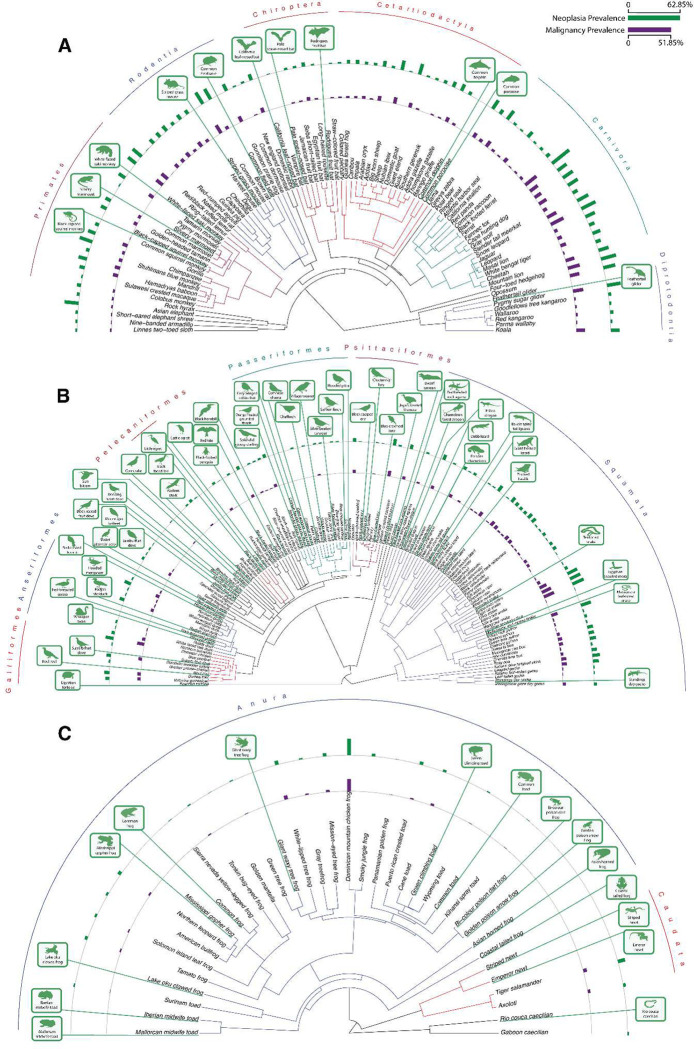
Neoplasia and malignancy prevalence across mammals (**A**), sauropsids (**B**), and amphibians (**C**). Silhouetted species indicated that zero neoplasms were reported.

**Fig. 2.**
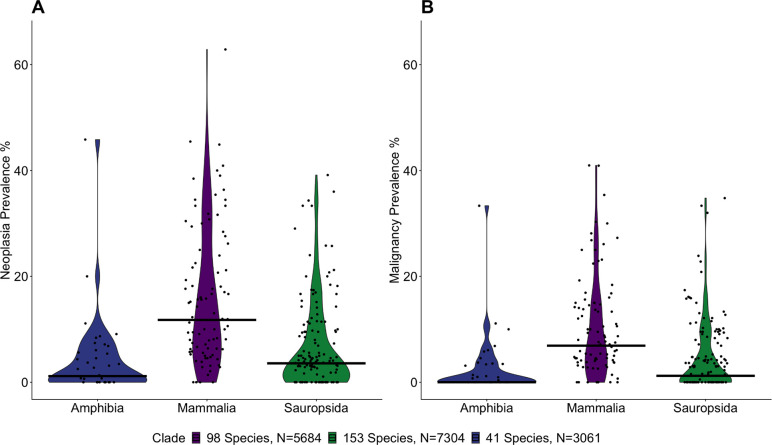
Distributions of of **A.** neoplasia (Kruskal-Wallace test: p = 2.906 × 10^−12^) and **B.** malignancy (Kruskal-Wallace test: p = 6.519 × 10^−11^) prevalences are different across three clades, Amphibia, Mammalia, and Sauropsida (Reptilia and Aves). Dots show the estimated species neoplasia prevalence and bars show the median for the clade. Neoplasia and malignancy prevalence for species were calculated by the proportion of the reported lesions among the total number of necropsies for that species.

We found evidence of neoplastic disease in necropsies across all analyzed taxonomic clades (Fig.1). Mean prevalence of neoplasia (mean = 9.07%, range = 0% - 62.86%) and malignancy (mean=5.91%, range=0%–40.95%) at death was highest in mammals (mean: Neoplasia= 15.28%, Malignancy = 9.82%; range: Neoplasia = 0% - 62.85%, Malignancy = 0% - 40.95%), followed by sauropsids (mean: Neoplasia=6.40% Malignancy=4.33%; range: Neoplasia= 0% - 39.13%, Malignancy: 0% - 34.78%) and amphibians (mean: Neoplasia = 4.16%; range: Neoplasia = 0% - 45.83%, Malignancy = 0% - 33.33%; Fig. 2), which confirms previous studies ^12,14^. Because reptiles are not a monophyletic clade, we have grouped them with birds in the sauropsida clade for the purposes of analysis. Despite a lower mean prevalence for both benign and malignant tumors, sauropsids and amphibians show a wide range of neoplastic disease burden across species. There is a small but highly statistically significant correlation between the prevalence of benign neoplasms and the prevalence of malignant neoplasms across species (*r*=0.2, *p*<0.0001, Fig. S64). Supplementary Tables ST1 and ST2 list the species with the highest and lowest neoplasia and malignancy prevalences, as well as the proportion of neoplasms that are malignant. Among the vertebrates with the highest prevalence of neoplasia, 63% of ferrets died with a neoplasm (45% of which was lymphoma), 56% of opposums died with a neoplasm (46% of which was in the lung), and 45% of hedgehogs died with a neoplasm (42% of which was in the alimentary tract) (ST3).

### Life History Analyses of Neoplasia Prevalence

**Fig. 3.**
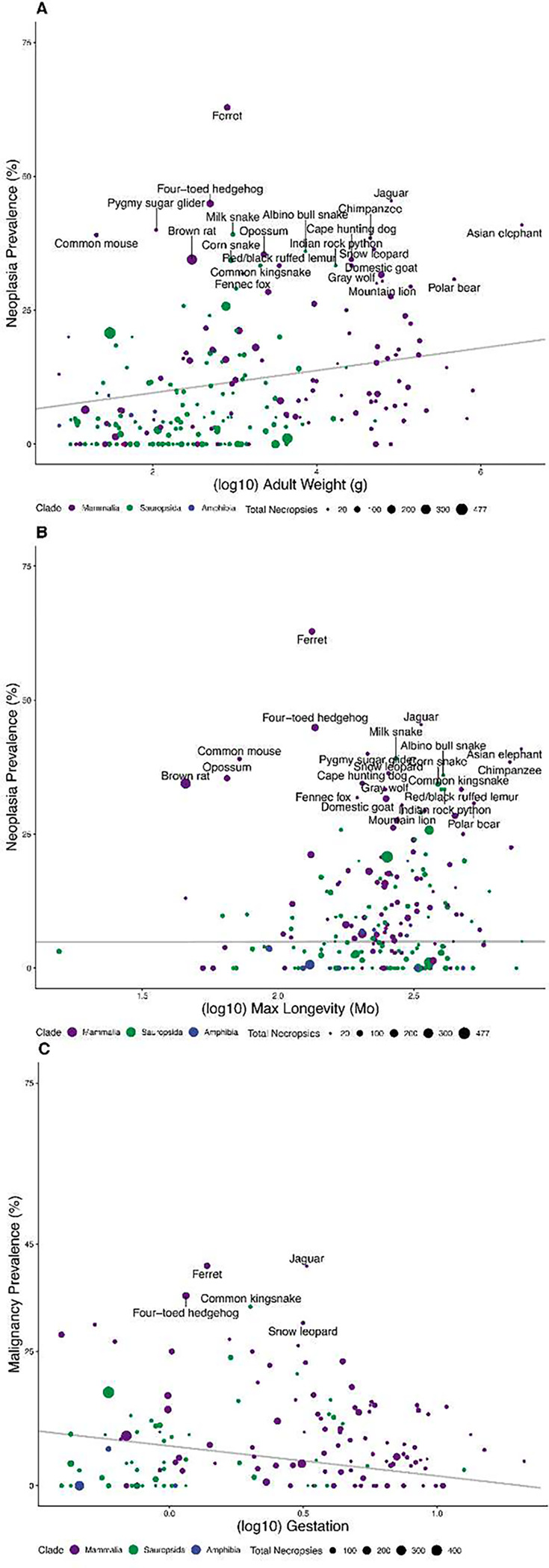
Significant life history factors associated with neoplasia and malignancy prevalence. **A.** Larger organisms have a higher neoplasia prevalence than smaller organisms (2.1% neoplasia per Log_10_g adult body mass, *p* = 0.007, R^2^ = 0.18, λ = 0.46). **B.** Longer lived organisms also have more neoplasia (0.01% neoplasia per Log_10_ month lifespan, p = 0.02, R^2^ = 0.16, λ = 0.34). **C.** Organisms with longer gestation times have a lower malignancy prevalence (−5.65% malignancies per Log_10_ months, *p* = 0.02, R^2^ = 0.01, λ = 0.41). When controlling for adult body mass, organisms with longer gestation times also have fewer neoplasias at death (−5.30% neoplasia per Log_10_ months, *p* = 0.1).

Evolutionary life history theory provides a framework for understanding the tradeoffs governing species’ survival and reproduction ^19,20^. Life history theory can be used to explain how species level traits shape organismal cancer risk based on trade-offs between investment in somatic maintenance (e.g., cancer suppression) and reproduction or growth. Several smaller studies have shown that specific life history traits can serve as prognostic indicators of neoplasia prevalence in animals managed under human care ^17,21^. We tested for relationships between life history factors and neoplasia or malignancy prevalence, controlling for phylogenetic relatedness, and weighting species data points by the number of necropsies in our dataset. In contrast to previous studies ^16–18^, we found an increase in neoplasia prevalence with increases in body mass (2.1% neoplasia per Log_10_g, *p* = 0.007; 0.65% malignancy per Log_10_g, *p* = 0.287) and maximum longevity (0.01% neoplasia per Log_10_g, *p* = 0.02; 0.0047% malignancy per Log_10_g, *p* = 0.276), not supporting Peto’s Paradox (Fig. 3). Animals with longer gestation times also get fewer malignancies (−5.56% malignancies with Log_10_ months, *p* = 0.02; Fig. 3C).

A multivariate model containing all significant predictors of neoplasia or malignancy (adult weight, maximum longevity, and gestation time) shows that both adult body weight (2.9% neoplasia per Log_10_g, *p* = 0.01) and gestation time (−18.6% neoplasia per month, *p* = 0.0001) provide independent information for estimating neoplasia prevalence. Because gestation time and adult weight are correlated (r = 0.50, *p* = 2.2 × 10^−16^), but have the opposite relationship to neoplasia and malignancy prevalence, we tested the two-variable model and found that when controlling for adult weight (3.8% neoplasia per Log_10_g, *p* = 0.0005), gestation time is also a significant predictor of neoplasia prevalence (−15.8% neoplasia per Log_10_ months, *p* = 0.001; R^2^ = 0.27), and vice versa. When controlling for gestation time, adult weight predicts malignancy prevalence, and when controlling for adult weight, gestation time also predicts malignancy prevalence (0.68% malignancies per Log_10_g, *p* = 0.0008; −6.61% malignancies per month of gestation, *p* = 0.0417; R^2^ = 0.198). Adult weight and gestation time were still statistically significant (adjusted *p*<0.05) predictors of neoplasia and malignancy prevalence after a 10% false discovery rate correction for multiple testing.

We found no evidence of a relationship between litter or clutch size and neoplasia prevalence (Figs. S21, S22). However, when we restrict the analysis to mammals, litter size is positively associated with both neoplasia and malignancy prevalence (neoplasia: *p* = 0.02, R^2^=0.55; malignancy: *p* = 0.03, R^2^ = 0.2; Suppl. Figs. 26 & 27), supporting our earlier analysis of 37 mammals from the San Diego Zoo ^17^. We also found that time to sexual maturity, growth rate and basal metabolic rates (which were only available for mammals) were not significant predictors of neoplasia or malignancy prevalence (Figs. S11, S12, S13, S14, S17, and S18). In addition to calculating the prevalence of neoplasms and malignancies, we also calculated the proportion of neoplasms that were malignant, which is a measure of the likelihood that a benign neoplasm transforms into a malignant one. We found no statistically significant relationships between any of those life history factors and the proportion of neoplasms that were malignant (Figs. S11, S13, and S17).

### DNA damage response and somatic mutation rates

**Fig. 4.**
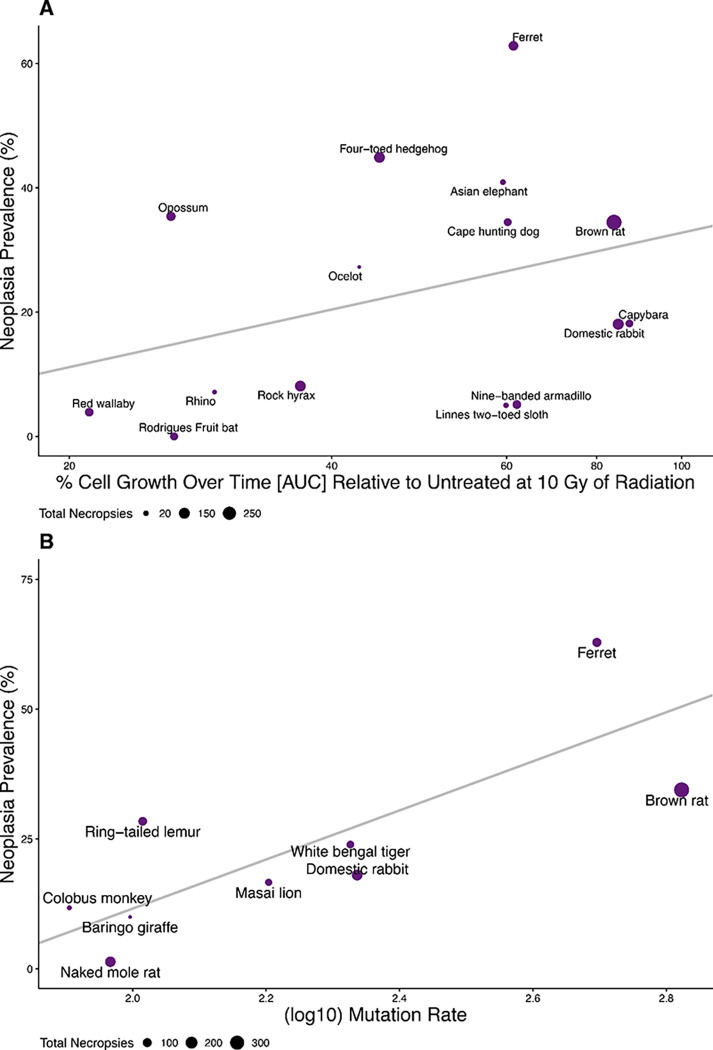
**A.** % Cell Growth Over Time [AUC] Relative to Untreated at 10 Gy of Radiation (plotted and analyzed on a Log_10_ scale) as a predictor of neoplasia prevalence in species’ fibroblast cell lines. (30.89% neoplasia per Log_10_ Cell Count Area Change, *p* = 0.22, R^2^ = 0.011, λ = 6.6 × 10^−5^) **B.** Log_10_ Mean Mutation Rate as a predictor of neoplasia prevalence (47.26% per single base substitution per genome per year, *p* = 0.0059, R^2^ = 0.96, λ = 1.00).

We tested primary fibroblasts from 15 species for their response to DNA damage (Fig. 4A; Suppl. Figs. 45–60). Mammalian cells have two primary mechanisms to address DNA damage: cell cycle arrest to allow for DNA repair and apoptosis ^16,22^. In a previous study, we documented that low prevalence of death from cancer in elephants was correlated with enhanced DNA damage response ^16^. Therefore, we hypothesized that DNA damage response would predict malignancy. Figure 4A shows the predicted trend for neoplasia prevalence, though they are not statistically significant. We also analyzed responses to lower doses of ionizing radiation and increasing doses of a chemotherapeutic drug (doxorubicin). No association with response and neoplasia or malignancy was observed (S45 - S60)

Evolving improved DNA damage response is one among a variety of possible mechanisms for reducing the somatic mutation rate. Because somatic mutations are responsible for causing many cancers^23–25^, we hypothesized that species with higher somatic mutation rates should have higher cancer prevalence. Somatic mutation rates were available for 9 of the species in our dataset, but even with that limited set, there is a clear positive association between somatic mutation rates and neoplasia prevalence (Figure 4B, pgls *p* = 0.0059). The trend is similar for somatic mutation rates and malignancy prevalence but it is not quite statistically significant (S59, pgls *p* = 0.087).

### Age as a Cancer Risk Factor in Animals

**Fig. 5.**
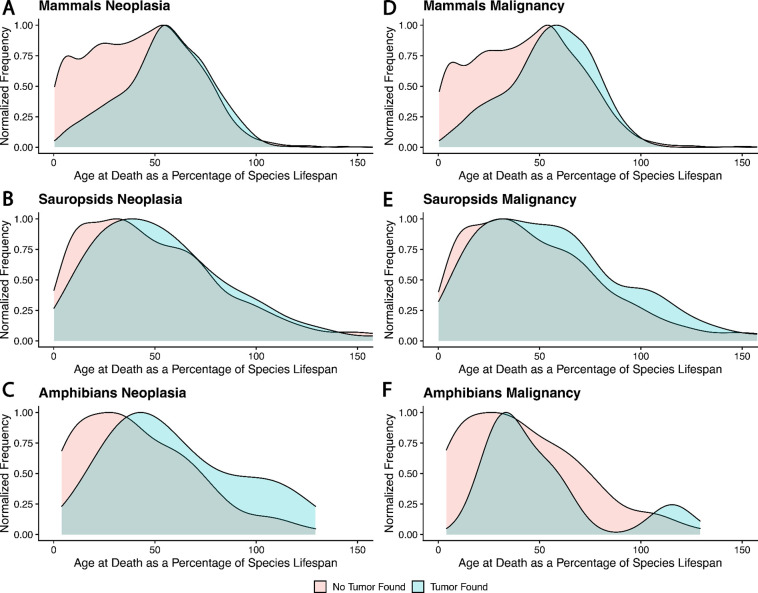
The density distribution of ages at death in animals with neoplasia versus non-neoplasia, adjusted for each species’ lifespan as specified in PanTHERIA. While the distributions of ages at death are different between necropsies showing neoplasia versus those that don’t (Two Sample Kolmogorov-Smirnov Test: Mammals: D=0.11, p =1.81 × 10^−6^; Sauropsids: D= 0.18, p = 4.48 × 10^−8^; Amphibians: D=0.5, p = 0.011), we found few neoplasias that could be explained by an organism living an extraordinarily long time in captivity, except in amphibians.

Age is the single biggest risk factor for the development of cancer in humans ^26^. Most mechanisms of somatic maintenance, including immune cell surveillance, DNA damage response, and telomere shortening, decrease in efficacy as we age ^27–31^. To test if observed neoplasms in animals under human care may be due to the animals living beyond their natural lifespans, we plotted the age of the animals with neoplasia at death, compared to the animals that died without neoplasias, scaled by their average lifespan (Fig. 5). The vast majority of animal deaths with neoplasia diagnoses occur before the average lifespan in most animals. Only amphibians seem to be developing more neoplasias as they live past their normal lifespan under human care(Fig. 5C). The distribution of tumor diagnoses across lifespan in these three clades also demonstrates that cancer is not limited to a disease solely of extended lifespan, and in sauropsids, neoplasia is not particularly a disease of old age (Fig. 5B).

## Phenotypic Models of Cancer Risk

### Evolution of cancer suppression and susceptibility

**Fig. 6.**
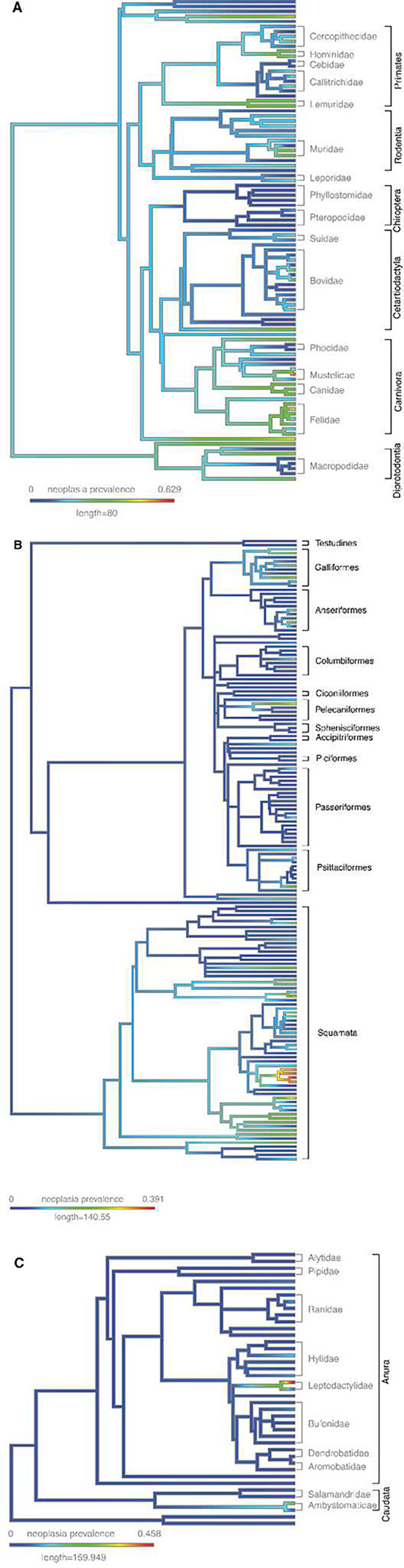
Cladogram depiction of cancer incidence within **A.** Mammals, **B.** Sauropsids, and **C.** Amphibians. Cladograms with the species labels at each tip can be found in Suppl. Fig. 63. Heat map coloration indicates relative prevalence of cancer within each branch, illustrating the diversity of neoplastic disease amongst closely related species. The scale is the same for each panel so that the differences between the clades are apparent.

Comparative phylogenetics provides a wealth of computational tools to model species’ trait evolution across a phylogeny ^32^. To explore how cancer susceptibility evolved across the tree of life (Fig. 6), we fit three of the most common phenotype evolution models (Ornstein-Uhlenbeck, Brownian Motion, and Early Burst) to neoplasia prevalence as a continuous trait. We found that a model of stabilizing selection on neoplasia prevalence (Ornstein-Uhlenbeck) fits the distribution of neoplasia prevalence the best (ST4). Malignancy prevalence evolution is also best explained by the Ornstein-Uhlenbeck model of sudden shifts followed by stasis in the phenotype.

## Methods

### Analysis of Veterinary Necropsy Records

We collected necropsy records with permission from 99 zoological institutions, aquariums, and other facilities that house animals under managed care. Gross necropsies had all been conducted by veterinary pathologists who specialize in nondomestic species and neoplasia was identified histologically by board-certified pathologists. Cases where suspect neoplasms were not examined histologically were excluded from the dataset. We used a terminology dictionary (ST5) to distinguish benign from malignant neoplasms based on the diagnoses in the necropsy reports. We excluded neonatal records to reduce bias from high levels of neonate and infant mortality that is common in many species. Because only common names were recorded for most records, we developed a tool, kestrel, to translate common names into scientific names which is available at https://pkg.go.dev/github.com/icwells/kestrel.

All of the institutions that provided prior approval for the use of their data in these analyses are Association of Zoos and Aquariums (AZA) accredited. AZA accreditation encourages the institution to perform a necropsy on all animals that die under their care to determine cause of death and to monitor morbidity and mortality of each species. Furthermore, each institution had IACUC approval with the Exotic Species Cancer Research Alliance (ESCRA) and the Arizona Cancer and Evolution Center (ACE) for the use of their deceased animal’s records of animals with neoplasia for use in this study. Previous analyses included both necropsies for animals diagnosed with neoplasia and animals that were still alive ^18^. In this study, we restricted our analyses to only necropsies - for both cancer and non-cancer diagnosed animals, because alive animals may harbor undetected cancer or might be eventually diagnosed with cancer, thus skewing estimates in cancer prevalence.

The data for our analyses are available in supplemental file 1.

### Comparative Phylogenetic Methods in Comparative Oncology

Interspecies comparisons must account for the shared ancestry and the constraint of natural selection on species’ traits before a determinant of any correlations can be made. For the life history models of neoplasia and malignancy prevalence, the R programming ^33^ packages “phytools” ^34^, “ape” ^35^, and “caper” ^36^ were all used for phylogenetic comparisons and the handling of phylogenetic data. To accomplish this we wrote the function *pglsSEyPagel* which is built upon phytool’s *pglsSEy* (phylogenetic generalized least squares for uncertainty in Y). pglsSEyPagel expands upon the pglsSEy function by adding the estimate of Pagel’s lambda ^37^ to the regression, rather than assuming it is fixed at 1 (i.e., Brownian motion).

### Testing for relationships with life history factors

We extracted data for maximum lifespan, adult body weight, basal metabolic rate, gestation length, litter size, time to sexual maturity, and growth rate from PanTHERIA ^38^. We used a weighted phylogenetic regression to control for non-independence of phenotypes (e.g. neoplasia prevalence) in closely related species. We report the phylogenetic signal, lambda, for each analysis, along with the p-value and R^2^. A single phylogenetic tree encompassing the three clades was collected from timetree.org. We pruned the tree to the 292 species in our data set using the setdiff and keep.tip/drop.tip functions in the APE R package. Estimates for neoplasia and malignancy prevalence are more accurate in species with more necropsies. To address the differences in number of necropsies, and to limit the noise from prevalence estimates based on few individuals, we weighted the species data points by the square-root of the number of necropsies records we have. Our R code for all analyses and figures included in this manuscript is freely available at https://github.com/zacharycompton/cancerAcrossVertebrates.git. In addition, we only analyzed species for which we had at least 20 necropsy results (previous studies had used 10 ^16^ or 20 ^17,18^ for the lower bound number of individuals). The main *pglsSEyPagel* analyses were done with all species together, including mammals, sauropsids and amphibians. In the analyses of litter size and gestation time, we also tested for a relationship with neoplasia prevalence in mammals alone. We carried out a total of 28 *pglsSEyPagel* analyses. To control for multiple testing, we used a false discovery rate (FDR) of 10%.

### DNA Damage Sensitivity Assays

Established, primary cells from mammals were obtained from San Diego Zoo Wildlife Alliance (Capybara, Linne’s Two Toed Sloth, Red Necked Wallaby, Rock Hyrax, Rodrigues Fruit Bat, Six Banded Armadillo, Southern White Rhino, and Virginia Opossum) or generated at Huntsman Cancer Institute from tissues collected from African Pygmy Hedgehog, Domestic Rabbit, Leopard, Asian Elephant, and Cape hunting dog, Brown rat (Cell Applications) and Normal Human Dermal Fibroblasts (Lonza) were commercially available. Detailed information on culture conditions, primary donor demographics, and passage numbers can be found in the supplementary information. Cells were seeded in 96-well plates at 2,000 cells per well in cell growth media and allowed to adhere overnight. The following day, doxorubicin was added at one of four concentrations (0μM [DMSO vehicle control], 0.11μM, 0.33μM, and 1μM). Each condition was tested in triplicate in three separate experiments. Cell proliferation and apoptosis were measured by real-time fluorescence microscopy (IncyCyte, Sartorius) at two-hour intervals for three days. Apoptosis was measured using a fluorescent cell death marker, Annexin V Dye (Sartorius). Images were processed and analyzed using IncuCyte software. The number of dead or dying cells were identified by counting Annexin V positive cells. In addition, cell count overtime was calculated using IncuCyte cell-by-cell software. To measure response to radiation-induced DNA damage, cells were irradiated with one of four doses: 0Gy, 0.4Gy, 2Gy, and 10 Gy. Radiation dose was delivered using an RS-2000 X-Ray Irradiator (Radsource). Cells were then seeded in 96-well plates in cell growth media containing Annexin V Dye (Sartorius). Cells were imaged by real-time fluorescence microscopy (IncuCyte, Sartorius) at two-hour intervals for five days. We estimated cell cycle arrest by normalizing the cell count of irradiated cells to untreated cells by dividing the area under the curve (AUC) of cell count over-time for treated cells by the AUC of cell count over time for the untreated (UT) cells. We converted that number into a percentage that represents the percent of cell proliferation relative to untreated cells. We then tested if this normalized amount of cell growth was predictive of neoplasia prevalence using the phylogenetically controlled *pglsSEyPagel* regression (Fig. 4A). Cagan et al. published somatic mutation rates (single base substitution per genome per year) for 16 species based on sequencing 208 individual colonic crypts from 56 animals from the London Zoo^39^. They divided the number of somatic mutations, detected by whole genome sequencing, by the age (in years) of the individual at the time that the tissue was taken, to estimate the mutation rate per year. Nine of those species are in our dataset, allowing us to use a pgls regression to test for an association between mean somatic mutation rates and neoplasia prevalence.

## Discussion

We estimated cancer prevalence across a wide range of tetrapod species that includes mammals, amphibians, reptiles and birds. Importantly, and contrary to previous studies, our analyses highlight limitations to Peto’s paradox, by showing that large animals do tend to get somewhat more neoplasms, and malignancies when controlling for gestation time, compared with smaller animals. This is particularly apparent when we control for the fact that animals with longer gestation times tend to get both fewer neoplasias and fewer cancers. However, large animals only get slightly more cancer than small animals. Whether or not they get as much cancer as one would expect from their body size and longevity depends on the model one uses to predict cancer prevalence as a function of body size ^40–42^. We hypothesize that animals with a longer gestation time than would be expected for their body size, may be investing more resources to control cell proliferation, and thereby reducing their vulnerability to cancer, compared to animals with shorter than expected gestation times. They may also prevent “jackpot” somatic mutations in gestation, which expand to large clones through the process of development and can significantly contribute to the risk of progressing to cancer^43,44^.

Cancer prevalence across species varies greatly. Here we have used the largest collection of species to date, and expanded our analyses beyond mammals ^16–18^, to test for patterns in cancer prevalence. We only include species with at least 20 necropsies (median 35), compared with 10 individuals per species in our original study ^16^, and weighted species more in our regression analyses if their cancer prevalence estimate is more accurate because it is based on more necropsies. This revealed adult weight and gestation time as significant predictors of neoplasia and malignancy prevalence. The fact that neoplasia prevalence seems to evolve by sudden shifts followed by stabilizing selection (the Ornstein-Uhlenbeck model of phenotypic evolution) is consistent with life history theory predictions that investment in somatic maintenance should be under selection in specific ecological conditions^19^, rather than drifting neutrally consistent with random Brownian motion. Some of the variation in cancer prevalence is still noise, due to estimating cancer prevalence from tens of individuals. However, much of that variation comes from the vast diversity of species across amphibians, reptiles, birds and mammals. We have explained only a small portion (~20%) of the variation in species vulnerability and suppression of cancer. There is clearly more to be discovered.

Peto’s Paradox is based on the expectation that large, long-lived animals should get more cancer because they have more cells that exist for a longer amount of time, increasing the likelihood that cancer will arise^10,11^. Although adult body weight is positively correlated with both neoplasia and malignancy prevalence, partially resolving Peto’s paradox, the effect size is much larger for neoplasia (3.8% neoplasia per Log_10_g) than for malignancies (0.68% malignancies per Log_10_g). There may be several explanations for this. The simplest being that malignancies are less common than neoplasias, which include both benign and malignant neoplasms. This reduces the statistical power and the expected size of the effects. However, the blunted relationship between body size and malignancy prevalence may also be due to natural selection acting to evolve mechanisms to suppress malignant transformation. Cancer suppression mechanisms are likely to have been under stronger selection among these larger and longer-lived organisms because it was more critical to suppress cancer for longer in order for these organisms to successfully reproduce. Thus, we might expect a relatively constant cancer rate across species with more cancer suppression mechanisms in large, long-lived organisms ^7,16,45–49^, and fewer in small, short-lived organisms.

Further, there are at least four transitions in neoplastic progression that natural selection might alter to increase the survivability of cancer in a species: 1) initiation of a neoplasm, 2) transformation of that neoplasm into malignancy (*i.e.*, invasion through the basement membrane), 3) metastasis, and 4) death caused by the cancer. Our data bear on the first two transitions. Specifically, we quantify the prevalence of neoplasms in a species, the prevalence of malignant neoplasms, and the proportion of neoplasms detected that are malignant. However, the selective pressure of cancer is ultimately due to its effects on mortality, and so quantifying the prevalence of cancer as a cause of death would be more relevant for evolutionary studies of comparative oncology ^18^.

The inclusion of cross-species functional assays highlighted in Fig. 4A demonstrates above all that there is tremendous variation in the cellular responses to radiation induced DNA damage. This is the first time functional assays to measure DNA damage response have been paired with species’ cancer prevalence. While response to DNA damage was not a significant predictor of neoplasia or malignancy prevalence at 4 or 10 grays, the trend follows our hypothesis that sensitivity to DNA damage may be one mechanism of cancer suppression ^16^. The variation observed in our DNA damage response assays suggests that many species may use other mechanisms of cancer suppression (such as immune surveillance or other forms of DNA damage response and somatic mutation suppression), thereby obscuring a simple relationship between our measurements and neoplasia or malignancy prevalence.

We found evidence of a connection between somatic mutation rates^39^ and neoplasia prevalence (Figure 4B). This is not surprising, given the role of somatic mutations in initiation^50–52^ and neoplastic progression^24,25^. Even with only 9 species in our analysis, a strong relationship between somatic mutation rate and neoplasia prevalence was already apparent. However, this relationship should be validated with the addition of both more somatic mutation rate data and more cancer prevalence data. Furthermore, future research should determine the mechanisms by which species have evolved low somatic mutation rates. This might include prevention of DNA damage, repair of DNA damage, removal of damaged cells, use of high fidelity DNA polymerases, or better mismatch repair^16,53–55^.

### Insights from comparative oncology for human cancers

Species with a high prevalence of particular cancers may help to generate targeted studies to elucidate the biological basis of those cancers, help draw informative parallels to particular cancer syndromes in humans, and serve as more realistic models for studying those cancers^56^. For instance 46% of the malignant tumors diagnosed in the opossum were lung adenocarcinomas (ST3), which is a leading cause of human cancer mortality in the United States ^57^. Hedgehogs may hold insights for colorectal cancer, the third leading cause of cancer mortality in the US, and ferrets may help us understand lymphoma. These spontaneous cancers may be more similar to human cancers than genetically manipulated mice, though that remains to be tested.

There is an exciting opportunity to discover the mechanisms for suppressing cancers in species with few to no observed neoplasms, or those that seem to prevent neoplasms from progressing to malignancy (Fig. 1). For example, the paucity of neoplasms in dolphins and porpoises may be due to a legacy of once having had large, long-lived cetacean ancestors that were under strong selection to suppress cancer ^45^. Our earlier analysis of cancer gene evolution in cetaceans found evidence of positive selection in a large number of tumor suppressor genes and proto-oncogenes^45^.

We previously found that animals that live longer than would be expected for their body size, like bats, tend to have more copies of tumor suppressor genes ^58^. In support of these observations, we find 9 bats, with an average lifespan of 16 years, have low neoplasia prevalence. We had hoped to discover species that are able to prevent malignant transformation by finding species that get a fair number of benign neoplasms, but few to no malignant neoplasms. The common paradigm in understanding the evolution of cancer suppression emphasizes the importance of protecting against tumor initiation. However, mechanisms that suppress malignant transformation could be similarly important in maintaining an organism’s fitness. Unfortunately, only a few species in our dataset fit that description. The species with the lowest proportion of malignant to benign neoplasms was the common squirrel, with only 12% of their tumors being malignant.

### Challenges for Comparative Oncology

There are a number of potential sources of bias in comparative oncology data. The protection against predation that zoological institutions offer fast life history animals may be extending their lifespan, and thereby unmasking a vulnerability to cancer at an age that they would never attain in the wild. However, Fig. 5 shows that the neoplasms were diagnosed prior to average lifespan in most cases, suggesting the extended lifespan due to managed care is not a large factor in these data. In fact, one surprise was that neoplasms appear in birds and reptiles at relatively young ages, although birds are known to be prone to virally induced cancers ^59^.

Our data results from the combination of the intrinsic cancer susceptibility of a species with the effect of the artificial conditions of managed populations, which is sometimes called an evolutionary mismatch ^60^. These animals were generally protected from predators, provided with veterinary care, had different diets and exercise from their wild counterparts, many lived in an urban environment, and interacted with different species and microbes than a free-ranging animal. It is striking to us that four of the species with the lowest prevalence of neoplasias in our dataset, the gray squirrel, the common dormouse, the striped grass mouse, and the common field vole are all from wild, urban populations. These necropsies come from the London Zoo which has a policy of performing a necropsy on any animal they recover that dies on its grounds, not just the animals under its care. This is a hint that cancer may well be less common in the wild, although this observation may be dependent on the species and their wildlife habitat^61^.

If the “gross” cause of death was obvious for an animal, an institution may not have submitted the animal’s samples for histopathology, and would not be included in our data collection. Similarly, if a particular type of neoplasia is difficult to detect in a necropsy (including some leukemias and intracerebral tumors), or was only present at a microscopic level, it may have been undercounted.

The functional assays were limited to fibroblast cell lines for the species for which we could obtain samples. Limited sample availability precluded the ability to control for biological factors such as age, which we expect to influence DNA damage sensitivity.

### The Future of Comparative Oncology

In the future, it will be important to collect additional data to validate our discoveries of species with particularly low and high cancer prevalence, such as those highlighted in Fig. 1. Several life history traits, such as basal metabolic rate, may explain cancer risk but with BMR measured in only a few species, we lacked statistical power to detect a relationship with neoplasia or malignancy prevalence. Here we have dramatically expanded the amount of data on cancer prevalence in non-human animals, but this must continue to be built upon if we are to match the robustness seen in human cancer epidemiology. In particular, much could be learned from analyzing the age-incidence curves of cancer ^18,62^, but that would require significantly more individuals for each species.

One of the most important holes in comparative oncology is cancer data on wild animals. Gathering data from free-ranging populations is challenging, as it is difficult to detect cancer due to the animals decaying or being eaten before they can be necropsied. Additionally, accurate age estimates are much more difficult to obtain in wild populations compared to those managed by humans. However, wild animal populations would greatly enhance the field of comparative oncology by validating species that have low cancer prevalence and testing for evolutionary mismatches for animals in captivity.

## Conclusion

Cancer is a problem of multicellularity ^1^. While we found a relationship between both body mass and gestation time with cancer prevalence, we are just beginning to discover patterns of cancer susceptibility and cancer defenses across species. It is likely that evolution has developed a variety of mechanisms for preventing cancer. The discovery of particular species with extremely low neoplasia prevalence provides opportunities for elucidating cancer suppression mechanisms that are compatible with life and reproductive success. Investigation of species with extreme vulnerability to a particular cancer may also help us understand those cancers as well as human syndromes that predispose to those cancers. We hope to learn from nature how to better prevent cancer in both humans and non-human animals.

## Data Availability

All data and code are available at zacharycompton/cancerAcrossVertebrates (github.com), with the exception of the ages of individual animals and the locations of their tumors, which are restricted due to privacy agreements with the contributing zoos. Access to that data may be granted with permissions of the zoos.
